# Multiple Myeloma and Renal Health: Navigating Selected Clinical Complexities

**DOI:** 10.7759/cureus.115585

**Published:** 2026-09-01

**Authors:** Azhar Hussain, Jack L Moniot, Mohsena Sumaya, David M Hughes

**Affiliations:** 1 Pharmaceutical and Biomedical Sciences, Touro University College of Pharmacy, New York, USA; 2 Pharmacy, Albany College of Pharmacy and Health Sciences, Albany, USA; 3 Hematology and Oncology, Pfizer (United States), Philadelphia, USA; 4 Medical Affairs, Pfizer (United States), Philadelphia, USA

**Keywords:** cast nephropathy, hypercalcemia-associated nephropathy, monoclonal gammopathy of undetermined significance (mgus), monoclonal immunoglobulin deposition disease, multiple myeloma, smoldering multiple myeloma (smm)

## Abstract

Multiple myeloma (MM) is a hematologic malignancy characterized by clonal plasma cell proliferation and is typically associated with excess monoclonal immunoglobulin production. MM results in multisystem complications that significantly impact morbidity and mortality if left unrecognized or improperly managed. Renal dysfunction is one of the most impactful complications and occurs through multiple mechanisms, requiring careful management of treatment complications, medication safety issues, and negative impacts on prognosis. This review examines the pathophysiology, diagnostic evaluation, pharmacologic management, and pharmacist-led supportive care strategies for MM-related renal dysfunction.

MM can result in renal injury through various mechanisms. The monoclonal immunoglobulin-related kidney diseases discussed in this review include cast nephropathy and monoclonal immunoglobulin deposition disease (MIDD). Cast nephropathy arises from the toxic accumulation of free light chains (FLCs) produced by clonal plasma cells. MIDD results in non-amyloid immunoglobulin deposition along various renal basement membranes. MM may also cause renal injury through non-immunoglobulin-related mechanisms, such as hypercalcemia-associated nephropathy (HAN). HAN results from elevated serum calcium levels and often occurs in patients with MM who have a high skeletal tumor burden. Early diagnosis requires prompt utilization of serum and urine testing, FLC assessment, imaging, and bone marrow evaluation.

Pharmacologic management must effectively control the disease while aiming for remission and ensuring patient safety. Factoring in complications such as renal impairment is critical to optimizing therapy. Bortezomib-based therapy, dose-adjusted immunomodulatory agents, careful use of bone-modifying therapies, hydration, avoidance of nephrotoxins, and electrolyte monitoring are essential components of care. Pharmacists are pivotal in identifying renal risk, adjusting medication regimens, preventing nephrotoxic exposure, educating patients, and coordinating multidisciplinary interventions. Early recognition and targeted management can improve or, in some cases, reverse renal complications, ultimately resulting in improved kidney function and patient outcomes in MM.

## Introduction and background

Multiple myeloma (MM) is a hematologic malignancy of clonal plasma cells that commonly infiltrate the bone marrow, leading to multisystem complications. Common early symptoms of MM include bone pain, fatigue, weight loss, and anemia. MM is distinct from other lymphomas and leukemias and can be identified by the presence of monoclonal proteins in the urine or serum. Although MM can affect anyone, it has a higher incidence in men and African Americans [1]. MM accounts for approximately 1% of all neoplastic diseases and is the second most common hematological malignancy in high-income countries, predominantly affecting the elderly population [2]. Within the United States, it is estimated that 36,000 new cases of MM will be diagnosed and 10,850 deaths will occur [3]. While MM remains incurable with current treatment options, advances in oncology have led to a broader range of therapeutics, with improvements in clinical outcomes, remission, and duration of survival.

Active MM is defined as clonal bone marrow plasma cells (BMPCs) ≥10% or biopsy-proven bony or extramedullary plasmacytoma and one or more of the CRAB criteria or myeloma-defining events (MDEs) [4]. MDEs are represented by the SLiM-CRAB criteria. The SLiM biomarkers include ≥60% clonal plasma cells on bone marrow examination; an involved/uninvolved serum free light chain (FLC) ratio ≥100, provided that the involved FLC level is ≥100 mg/L; or more than one focal lesion on MRI, with each lesion measuring ≥5 mm. The CRAB criteria include hypercalcemia, renal insufficiency, anemia, and bone lesions. These criteria can guide the diagnosis and clinical assessment of patients with MM to support optimal care [5]. Among these, renal dysfunction is one of the most serious and common complications of MM, with approximately 50% of patients with MM experiencing chronic kidney disease (CKD) or acute kidney injury (AKI) at some point during the course of their disease. In severe cases, renal dysfunction may progress to end-stage kidney disease requiring dialysis, which has been reported in approximately 10% of patients [2]. Some of the most common mechanisms of renal injury associated with monoclonal proteins include intratubular cast formation, direct tubular toxicity, monoclonal immunoglobulin deposition disease (MIDD), and hypercalcemia [2].

This narrative review examines the pathophysiology, diagnostic evaluation, pharmacologic management, and pharmacist-led supportive care strategies for MM-related renal dysfunction. In MM, renal impairment is associated with an increased risk of treatment toxicity and earlier mortality following diagnosis. The risk of death for patients with severe renal impairment may be fourfold higher than that for patients with either moderate or no renal impairment [6]. Renal impairment also increases the risk of toxicity from medications required for treatment. Medications that are essential for treating MM can be either directly or indirectly nephrotoxic and require careful monitoring and vigilant oversight. Because renal dysfunction can affect both mortality and medication regimens in patients with MM, early detection and intervention are pivotal.

Given the complexities of MM treatment, pharmacists play a crucial role in optimizing patient care and support. Pharmacists have a broad range of responsibilities, including interpreting renal laboratory values, adjusting medications to prevent nephrotoxic effects, and providing consultation on medication adherence and supportive care. Pharmacists are integral members of multidisciplinary care teams and are equipped to detect early signs of renal insufficiency, helping to ensure that an MM patient's medication regimens are both effective and safe. While subsequent sections will explore specific interventions and strategies that pharmacists implement to optimize care, it is essential to highlight that their involvement within a multidisciplinary care team is crucial to achieving optimal patient outcomes.

Although not the focus of this review, amyloid light-chain (AL) amyloidosis is important to acknowledge as a distinct diagnosis in patients with MM experiencing renal impairment. AL amyloidosis causes extracellular deposition of Congo red-positive fibrils in tissues. AL amyloidosis can lead to asymptomatic proteinuria or nephrotic syndrome and can be characterized as a systemic disorder [7]. Although the management of AL amyloidosis overlaps with that of MM, it requires distinct diagnostic criteria and strategies, along with different therapeutic approaches.

The search strategy for this narrative review consisted of a comprehensive search of multiple resources, including PubMed and UpToDate, as well as Google Scholar and the National Comprehensive Cancer Network and International Myeloma Working Group guidelines. The literature search was conducted from May 2025 through May 2026, using search terms including “multiple myeloma,” “renal impairment,” “renal dysfunction,” “kidney disease,” “acute kidney injury,” “chronic kidney disease,” “cast nephropathy,” “light-chain cast nephropathy,” “free light chains,” “light chain nephrotoxicity,” “monoclonal immunoglobulin deposition disease,” “hypercalcemia,” “hypercalcemia-associated nephropathy,” “renal recovery,” “dialysis,” “high-cutoff hemodialysis,” “pharmacologic management,” “supportive care,” “nephrotoxicity,” “renal dose adjustment,” and “pharmacist intervention.” No strict publication date restriction was applied to the search methods. However, preference was given to literature published within the past 20 years, with older, directly relevant publications included when necessary. The literature selected for inclusion was published in English. References were selected through author review of the identified literature, with preference given to recent publications on randomized trials, primary studies, clinical guidelines, and other publications directly relevant to the objectives of this review. Publications that were not relevant to renal manifestations or management strategies were excluded. AL amyloidosis was excluded from the review because it requires a distinct clinical diagnosis with separate criteria and management considerations. Because this is a narrative review, a risk-of-bias assessment was not performed, and the literature selection process may be subject to selection bias.

This article was presented by the authors as poster presentations at the 2025 NCODA Fall Summit and the 2025 ASHP Midyear Clinical Meeting.

## Review

Pathophysiology of MM

MM is a hematological malignancy characterized by the clonal proliferation of plasma cells within the bone marrow. This proliferation results from primary genetic events, such as hyperdiploidy, marked by trisomies of odd-numbered chromosomes, and non-hyperdiploid translocations involving the immunoglobulin heavy chain (IgH) locus at 14q32 [8]. Notable translocations include t(11;14)(q13;q32), leading to cyclin D1 (CCND1) overexpression; t(4;14)(p16;q32), affecting fibroblast growth factor receptor 3 (FGFR3) and multiple myeloma SET domain (MMSET); and t(14;16)(q32;q23), involving musculoaponeurotic fibrosarcoma oncogene (MAF) gene overexpression [8,9]. These primary events often occur during class-switch recombination or somatic hypermutation in germinal centers, setting the stage for monoclonal gammopathy of undetermined significance (MGUS) [10,11].

MGUS is a premalignant condition characterized by clonal BMPCs comprising <10% of marrow cells, serum monoclonal protein <3 g/dL, and the absence of MDEs (SLiM-CRAB) or AL amyloidosis [12]. This premalignant condition can progress to MM with the evolution of genetic abnormalities. Chromosome 13 deletions, observed in approximately 50% of patients with MM, may occur as early or progression-associated events depending on the molecular subtype. Other abnormalities include activating mutations in the Rat Sarcoma viral oncogene homolog (RAS) pathway found in approximately 40% of newly diagnosed MM patients, Myelocytomatosis oncogene (MYC) dysregulation associated with advanced intramedullary disease and extramedullary progression, tumor suppressor inactivation, specifically p16 and p53, linked to extramedullary disease, and gain(1q) along with del(1p32) [13]. These molecular transformations may contribute to progression from MGUS and SMM to MM. However, progression is heterogeneous and does not invariably follow a clinically observed linear sequence [12,14].

SMM is a clinically distinct entity from MGUS in terms of diagnostic criteria, progression risk, and management. SMM requires close monitoring, typically every 3 to 6 months, for signs of disease progression, including MDEs defined by the SLiM-CRAB criteria [15].

The 2/20/20 risk stratification model, developed by the International Myeloma Working Group (IMWG), estimates a patient’s risk of progression from SMM to active MM. It uses three measurable factors [16]: (1) M-protein >2 g/dL; (2) BMPCs >20%; and (3) FLC ratio >20.

Based on the number of these risk factors, patients are classified into: (A) Low-risk (0 risk factors): ~6% chance of progression to MM within 2 years; (B) Intermediate-risk (1 factor): ~18% chance of progression within 2 years; and (C) High-risk (≥2 factors): ~44% chance of progression within 2 years.

This stratification is used to guide surveillance intensity and to identify patients who may benefit from early therapeutic intervention [14,15].

The diagnostic and clinical distinctions among MGUS, SMM, and active MM are summarized in Table 1.

**Table 1:** Classification of plasma cell disorders.

Disorder	Criteria	Clinical Presentation	Treatment
Monoclonal Gammopathy of Undetermined Significance (MGUS)	Clonal bone marrow plasma cells comprising <10% of marrow cells, serum monoclonal protein <3 g/dL, and the absence of myeloma-defining events (SLiM-CRAB) or AL amyloidosis.	Asymptomatic	Observation
Smoldering Multiple Myeloma (SMM)	Serum monoclonal protein ≥3 g/dL or urinary monoclonal protein ≥500 mg per 24 h and/or clonal bone marrow plasma cells 10% to <60%. Absence of myeloma-defining events or amyloidosis.	Asymptomatic	Observation/monitoring; treatment may be considered for high-risk SMM
Multiple Myeloma (MM)	Clonal bone marrow plasma cells ≥10% or biopsy-proven bony or extramedullary plasmacytoma and ≥1 myeloma-defining event, including ≥60% clonal plasma cells on bone marrow examination; an involved/uninvolved serum free light chain ratio ≥100, provided that the involved free light chain level is ≥100 mg/L; more than one focal lesion on MRI, with each lesion measuring ≥5 mm; or a CRAB feature.	May present with CRAB manifestations and/or meet biomarker criteria for active MM	Myeloma therapy + supportive care

The bone marrow plays a significant role in the pathogenesis of MM, driven by inflammatory mediators, cellular interactions, and cytokines. The bone marrow contains mesenchymal stromal cells (MSCs) that can contribute to progression to MM by releasing interleukin-6 (IL-6), leukemia inhibitory factor (LIF), and C-C motif chemokine ligand 2 (CCL2), which stimulate malignant plasma cell growth [17]. The production of IL-6, C3, annexin A1 (ANXA-1), and vascular endothelial growth factor A (VEGF-A) influences the myeloid cell compartment. This pro-tumorigenic signaling intensifies with the conversion of MSCs into an inflammatory phenotype (iMSCs), triggered by interleukin-1 (IL-1) produced by monocytes, tumor necrosis factor (TNF) released by natural killer (NK) and CD8+ lymphocytes, and damage-associated molecular patterns (DAMPs) from tumor-derived exosomes. These iMSCs not only support the growth of malignant plasma cells but also remodel the myeloid compartment by secreting IL-6, C3, ANXA-1, and VEGF-A, thereby creating an immunosuppressive niche. This cytokine-rich microenvironment, characterized by elevated levels of IL-6, IL-1β, B-cell-activating factor (BAFF), and a proliferation-inducing ligand (APRIL), enhances myeloma cell survival, proliferation, and resistance to apoptosis. As the disease progresses, severe immune dysregulation occurs, characterized by the expansion of myeloid-derived suppressor cells (MDSCs) and M2 macrophages, alongside a shift in T cell populations toward the immunosuppressive T helper 17 cells (Th17) and Treg phenotypes, while cytotoxic CD8+ and effector CD4+ T cell activities are inhibited, facilitating immune evasion and disease progression [18,19].

The uncontrolled proliferation of clonal plasma cells and the excessive production of monoclonal immunoglobulins can lead to end-organ damage in multiple systems, resulting in renal complications, peripheral neuropathy, cardiac complications, recurrent infections, bone complications, and anemia [20], as shown in Figure 1.

**Figure 1:**
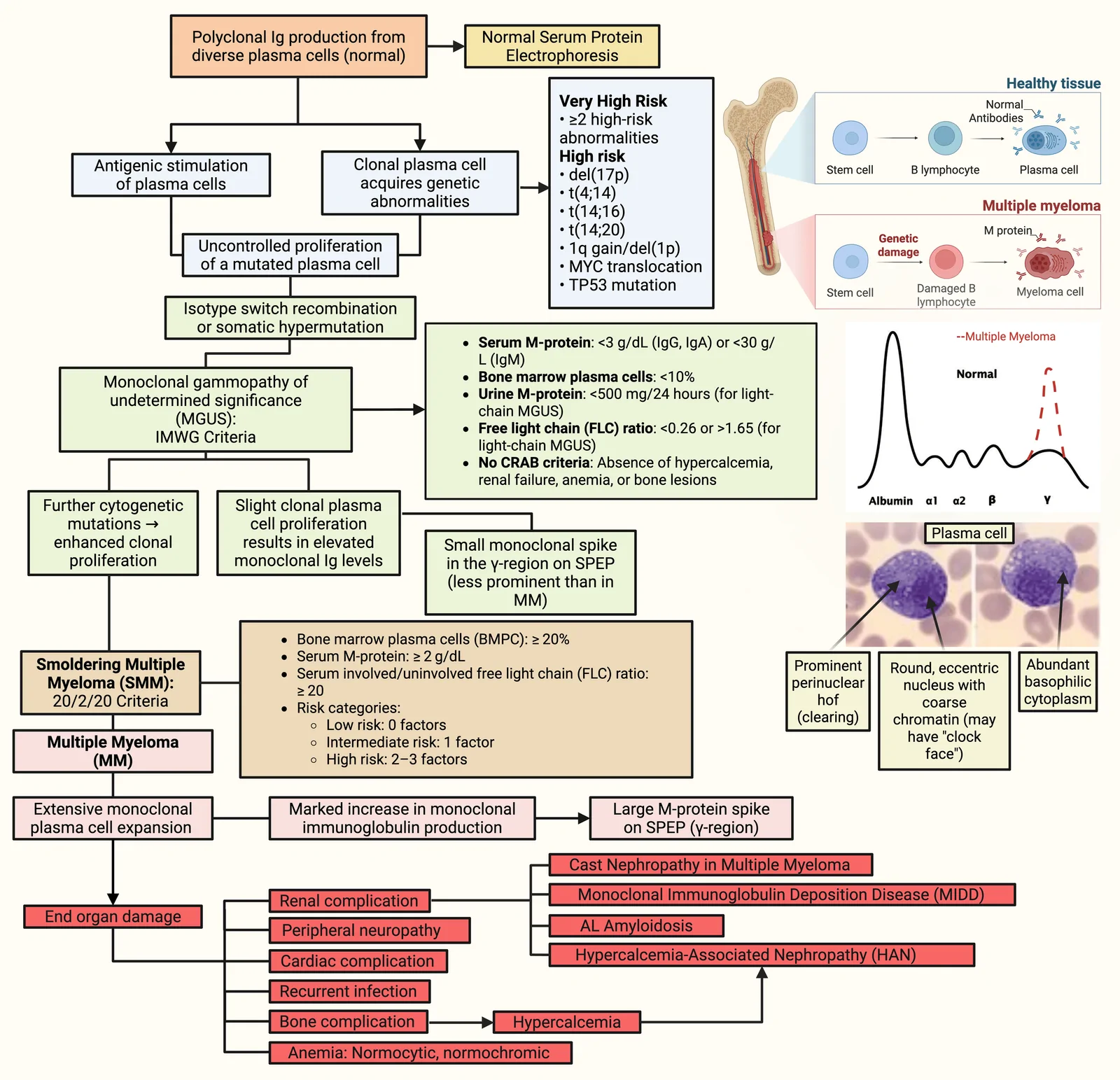
Pathophysiology of multiple myeloma. This figure visually illustrates the progression from normal polyclonal immunoglobulin production to the uncontrolled proliferation of clonal plasma cells associated with MGUS, SMM, MM, and ultimately, end-organ damage involving multiple systems. MM: Multiple myeloma; MGUS: Monoclonal gammopathy of undetermined significance; SMM: Smoldering multiple myeloma; CRAB: Hypercalcemia, renal insufficiency, anemia, and bone lesions. Image credits: Azhar Hussain, Jack L. Moniot, Mohsena Sumaya, David M. Hughes. Created with BioRender.com. This figure is an original author-created illustration. Artificial intelligence was not used in the creation of this figure.

This literature review focuses on renal complications associated with MM, including cast nephropathy, hypercalcemia-related nephropathy, and MIDD. These conditions represent some of the most prevalent and clinically relevant mechanisms by which MM causes kidney dysfunction [20]. Although clonal plasma cell disorders may result in AL amyloidosis, this article does not address that topic. AL amyloidosis is characterized by a distinct pathogenesis and renal presentation, often marked by nephrotic-range proteinuria with minimal tubular obstruction, making it a unique clinical entity that requires separate discussion.

Cast nephropathy in MM

Cast nephropathy, also referred to as myeloma kidney, is the most prevalent renal complication associated with MM and represents a significant cause of morbidity in affected patients. Cast nephropathy is seen in over 50% of patients who died with MM and renal involvement and in 40% to 60% of renal biopsies from living patients with the same complication [21]. It arises from the toxic accumulation of FLCs produced by clonal plasma cells.

In MM, the neoplastic proliferation of plasma cells leads to an overproduction of monoclonal immunoglobulin light chains. These FLCs circulate within the bloodstream and undergo glomerular filtration. Under normal physiological conditions, small quantities of FLCs are reabsorbed and degraded by the proximal tubules. Nevertheless, in the context of myeloma, excessive FLCs surpass the reabsorptive capacity of proximal tubular cells and continue further into the distal nephron [21,22].

In the distal tubules and collecting ducts, FLCs interact with Tamm-Horsfall glycoprotein (uromodulin), resulting in the formation of insoluble proteinaceous casts. These casts obstruct tubular flow, cause direct damage to the tubular epithelium, and induce interstitial inflammation. The combined effects of mechanical obstruction, tubular dilation, and cellular injury culminate in AKI. If unrecognized or inadequately managed, cast nephropathy may progress to irreversible renal failure and end-stage renal disease (ESRD). Clinically, patients may present with non-nephrotic proteinuria, electrolyte disturbances, and a rapid decline in renal function. Diagnosis is often confirmed through a kidney biopsy, which reveals intratubular casts and associated inflammation. Early recognition and prompt reduction of the FLC burden, typically achieved through anti-myeloma therapy, are essential for reducing renal injury and improving long-term outcomes [7,21,22]. The major steps in this process, including FLC overproduction, tubular cast formation, obstruction, inflammation, and progression to kidney injury, are summarized in Figure 2.

**Figure 2:**
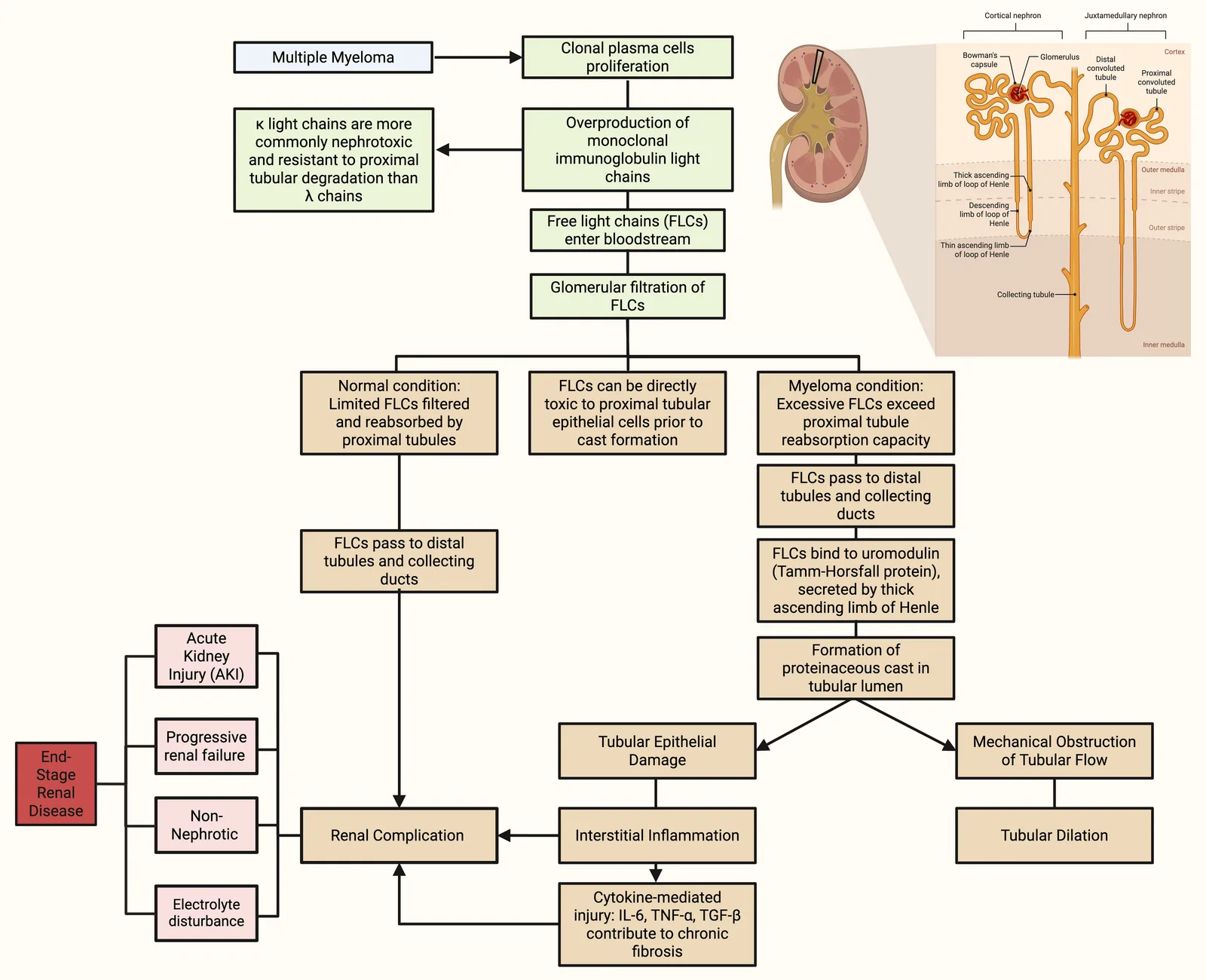
Cast nephropathy in multiple myeloma. This illustration depicts the pathogenesis of cast nephropathy, resulting in renal complications such as AKI, progressive renal failure, electrolyte disturbances, and end-stage renal disease. FLCs: Free light chains; AKI: Acute kidney injury; IL-6: Interleukin-6; TNF-α: Tumor necrosis factor-alpha; TGF-β: Transforming growth factor-beta. Image credits: Azhar Hussain, Jack L. Moniot, Mohsena Sumaya, David M. Hughes. Created with BioRender.com. This figure is an original author-created illustration. Artificial intelligence was not used in the creation of this figure.

Hypercalcemia-associated nephropathy (HAN)

HAN represents the second most prevalent cause of renal failure in patients with MM; hypercalcemia occurs in approximately 30% of individuals and creates a complex pathophysiological cascade that requires prompt recognition and intervention [23]. Hypercalcemia predominantly occurs in patients with myeloma who possess the highest skeletal tumor burden; however, it may also indicate the overall extent of disease, irrespective of serum parathyroid hormone-related protein (PTHrP) status. The underlying causes remain uncertain; however, they may be associated with the extent of osteoclastic activity generated by myeloma cells and the status of glomerular filtration [24].

The pathogenesis begins with a fundamental disruption of bone homeostasis typical of MM, in which clonal plasma cells infiltrate the bone marrow and create a highly imbalanced microenvironment by increasing RANKL levels and decreasing osteoprotegerin levels [24]. This molecular dysregulation stimulates osteoclast activity while inhibiting osteoblast function, leading to typical osteolytic lesions and substantial release of calcium from the bone matrix into the bloodstream. Unlike other causes of hypercalcemia, myeloma-related bone destruction can be extensive and rapid, especially in patients with widespread skeletal involvement, often raising serum calcium levels above 11 mg/dL and initiating a series of renal complications [24,25].

Elevated calcium levels have direct toxic effects on the kidneys through multiple pathways. Calcium deposits in the renal tubules promote nephrocalcinosis, with deposits accumulating in the tubules and interstitium, leading to chronic tubulointerstitial inflammation and progressive fibrosis. High calcium concentrations within the renal tubules can promote calcium precipitation, resulting in crystalline deposits that physically damage the normal structure of the tubules. Hypercalcemia also causes significant changes in renal blood flow through vasoconstriction of the afferent arteriole, resulting in reduced renal blood flow and glomerular filtration rate. A particularly harmful aspect is that hypercalcemia disrupts the kidney’s ability to concentrate urine by interfering with antidiuretic hormone action in the collecting duct. This disturbance leads to nephrogenic diabetes insipidus, characterized by polyuria, polydipsia, and an inability to properly concentrate urine. Paradoxically, although increased urine production might be expected to increase calcium excretion, it actually causes volume depletion and prerenal azotemia. The resulting volume contraction worsens hypercalcemia by decreasing calcium clearance, creating a dangerous positive feedback loop [24-26].

The most clinically significant aspect is the tendency to form self-sustaining cycles of deterioration. Declining GFR, due to vasoconstriction and structural damage, reduces calcium excretion, worsening hypercalcemia. Systemic effects such as nausea, vomiting, mental status changes, and decreased oral intake further contribute to volume depletion, particularly when nephrogenic diabetes insipidus is present. This cycle is especially dangerous in myeloma patients, who often have additional complications such as light chain cast nephropathy or MIDD. These conditions accelerate progression from AKI to CKD by decreasing GFR, thereby increasing FLC levels in the tubular lumina and reducing flow rates, which promotes cast formation. Recognizing this pathogenesis is crucial for clinical management. It underscores that this is a medical emergency requiring immediate intervention, including aggressive hydration when possible and prompt correction of hypercalcemia with carefully chosen calcium-lowering agents based on renal function [25,26].

Understanding these mechanisms highlights the importance of early detection and aggressive treatment to preserve kidney function. Effective management involves breaking the cycle promptly through coordinated supportive care and targeted anti-myeloma therapy. Insights from studying this complication have wider implications for managing calcium disorders in other cancers. They reveal the complex links between bone metabolism and kidney health in blood cancers, emphasizing a multidisciplinary approach. Hypercalcemia can accelerate renal damage by promoting light chain cast formation and increasing inflammation in the tubules, ultimately causing rapid progression from mild AKI to CKD or end-stage renal failure if structural damage, such as nephrocalcinosis and fibrosis, occurs [24,25]. The renal effects of MM-related hypercalcemia, such as tubulointerstitial damage, vasoconstriction, structural damage, and, consequently, ESRD, are summarized in Figure 3.

**Figure 3:**
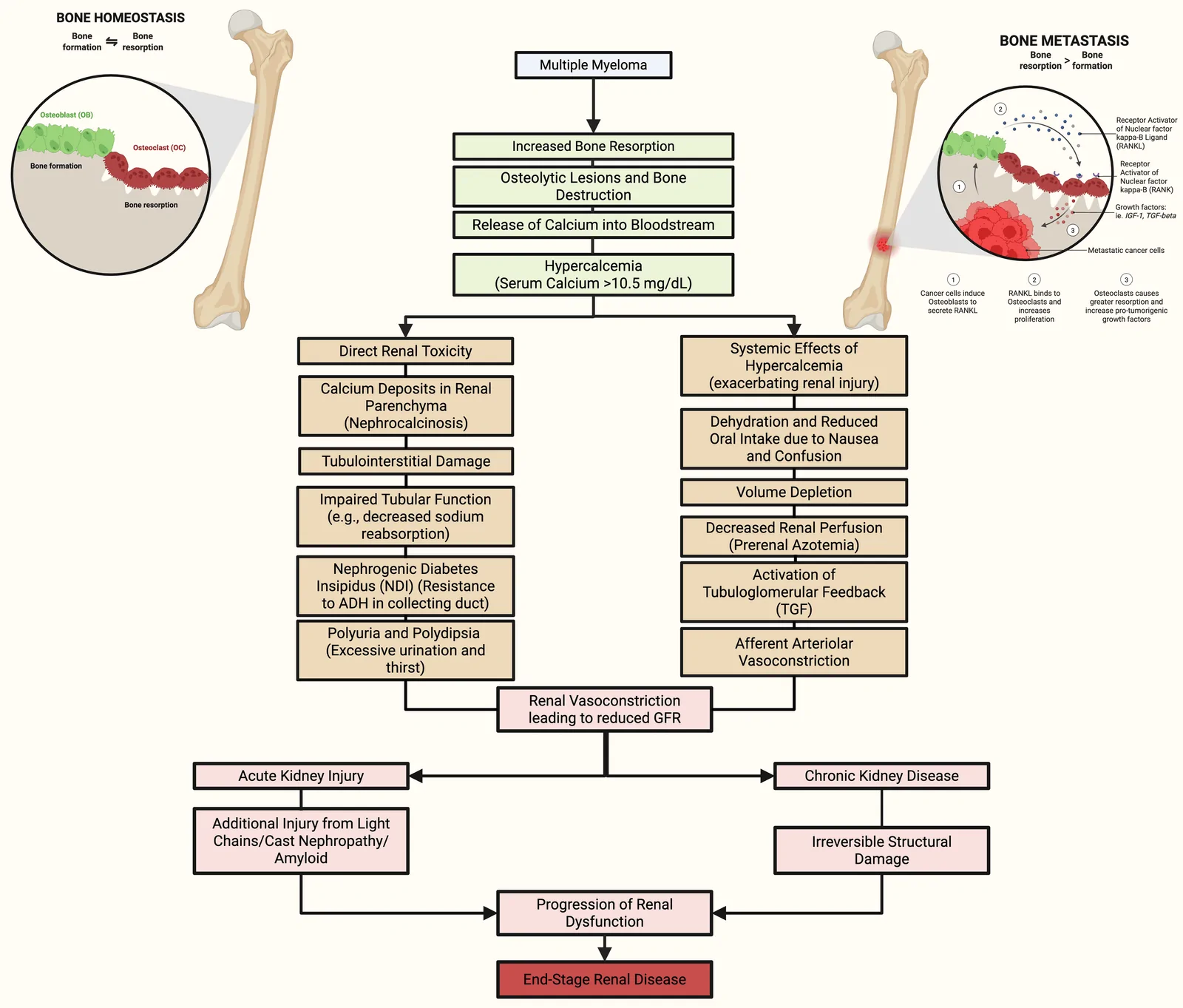
Hypercalcemia-associated nephropathy (HAN) in multiple myeloma. This illustration depicts how hypercalcemia-associated nephropathy leads to renal damage and dysfunction. ADH; Antidiuretic hormone; NDI: Nephrogenic diabetes insipidus; GFR: Glomerular filtration rate; TGF: Tubuloglomerular feedback. Image credits: Azhar Hussain, Jack L. Moniot, Mohsena Sumaya, David M. Hughes. Created with BioRender.com. This figure is an original author-created illustration. Artificial intelligence was not used in the creation of this figure.

MIDD

MIDD is a rare yet serious complication associated with plasma cell dyscrasias and may also occur within the spectrum of monoclonal gammopathy of renal significance (MGRS). It is characterized by the non-amyloid deposition of monoclonal immunoglobulin fragments, predominantly light chains, along renal basement membranes. MIDD is defined by the linear deposition of monoclonal immunoglobulin (MIg) along renal basement membranes [27].

MIDD arises from the proliferation of clonal plasma cells, leading to the systemic release of monoclonal immunoglobulins into the bloodstream. The immunoglobulins may comprise light chains alone (light chain deposition disease [LCDD], the predominant subtype, accounting for approximately 80% of MIDD), heavy chains (HCDD), or both light and heavy chains (LHCDD). In contrast to amyloidosis, deposits in MIDD are Congo red-negative and appear as granular, powdery material when observed under electron microscopy [27,28].

Renal deposition predominantly occurs along the glomerular basement membrane (GBM) and tubular basement membrane (TBM), as well as in the interstitium and blood vessels. The involvement of the glomeruli is nearly ubiquitous, with 60% of LCDD cases exhibiting nodular glomerulosclerosis. Tubular involvement is also significant, characterized by linear PAS-positive deposits, while interstitial alterations include extracellular matrix accumulation and fibrosis. These structural changes result in clinical manifestations such as proteinuria, renal insufficiency, hypertension, and variable hematuria [27].

Subtypes of MIDD exhibit distinct prognoses. Pure LCDD correlates with a more favorable outcome. Conversely, HCDD is associated with frequent occurrences of nephrotic syndrome and abnormalities such as deletion of the CH1 domain. LHCDD presents with mixed deposition and demonstrates an intermediate prognosis. The combination of LCDD with cast nephropathy is associated with the poorest renal prognosis, often advancing rapidly to ESRD. Early diagnosis via renal biopsy and prompt initiation of anti-myeloma therapy are essential to preserve renal function and improve long-term survival in patients with MIDD [27,29,30]. The renal deposition in kidney structures, structural damage, clinical manifestations, and prognostic differences among MIDD subtypes are illustrated in Figure 4.

**Figure 4:**
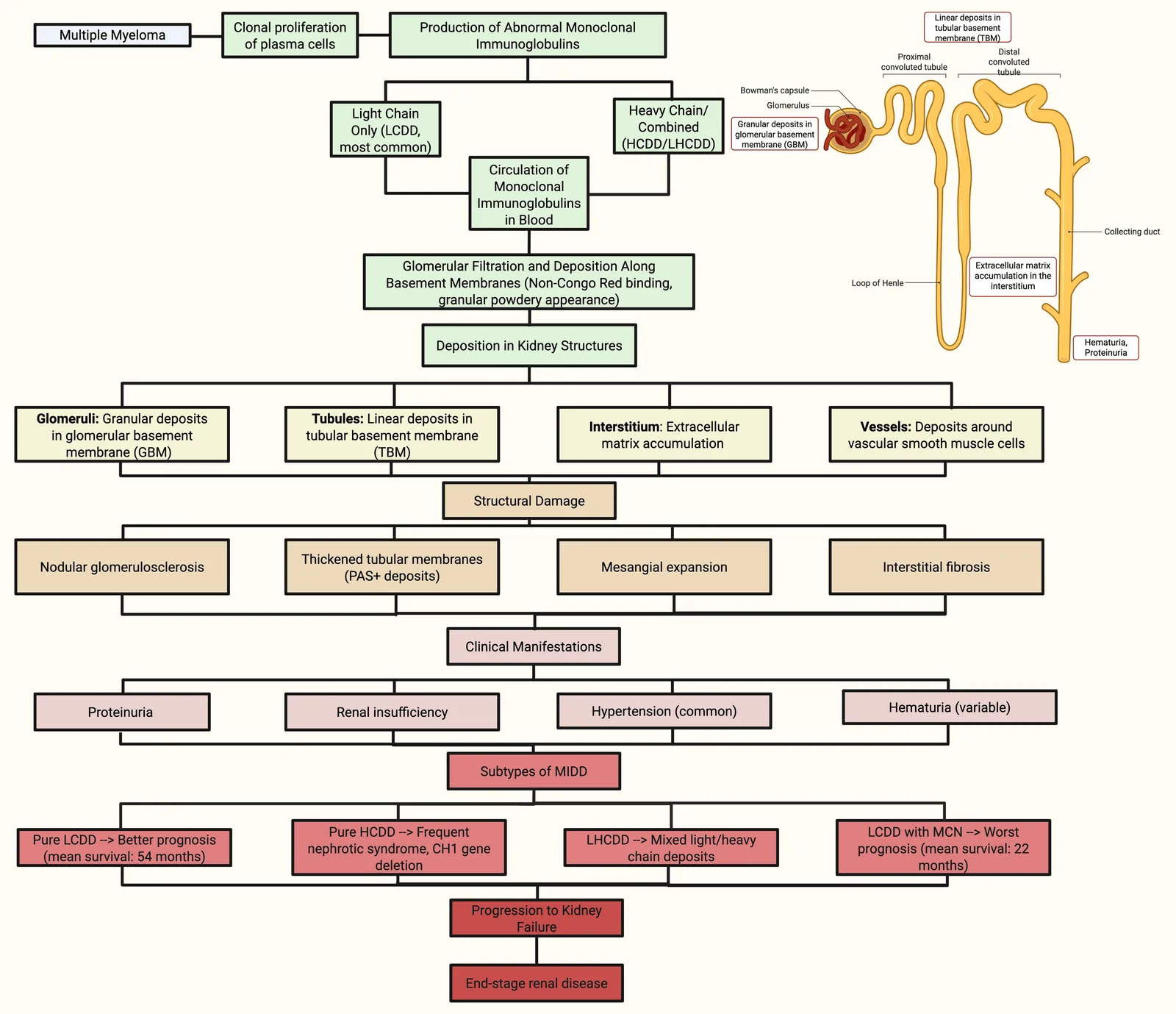
Monoclonal immunoglobulin deposition disease (MIDD). This illustration depicts the progression of monoclonal immunoglobulin deposition disease, including structural damage, clinical manifestations, and the different subtypes that can present. MIDD: Monoclonal immunoglobulin deposition disease; LCDD: Light chain deposition disease; HCDD: Heavy chain deposition disease; LHCDD: Light and heavy chain deposition disease; GBM: Glomerular basement membrane; TBM: Tubular basement membrane; MCN: Myeloma cast nephropathy; PAS: Periodic acid-Schiff. Image credits: Azhar Hussain, Jack L. Moniot, Mohsena Sumaya, David M. Hughes. Created with BioRender.com. This figure is an original author-created illustration. Artificial intelligence was not used in the creation of this figure.

Diagnostic criteria

The diagnostic process for MM typically begins with clinical suspicion, often triggered by symptoms such as bone pain, fatigue, recurrent infections, or incidental laboratory findings, including anemia, hypercalcemia, or elevated total protein levels. The initial evaluation includes comprehensive blood and urine tests, such as serum protein electrophoresis (SPEP) to identify monoclonal protein spikes, immunofixation to determine the monoclonal protein subtype, and serum FLC assays to detect light chain disease. Urine protein electrophoresis (UPEP) is used to detect Bence-Jones proteins. Routine laboratory investigations assess renal function, calcium concentrations, and the presence of cytopenias. Imaging modalities, such as whole-body low-dose CT, PET-CT, MRI, or skeletal survey, facilitate the identification of osteolytic lesions or bone marrow infiltration, which are characteristic features of myeloma [31].

The subsequent step involves bone marrow aspiration and biopsy to quantify clonal plasma cells when laboratory studies are suggestive of myeloma. Cytogenetic and molecular analyses of the marrow sample, including fluorescence in situ hybridization (FISH) and karyotyping, assist in defining prognosis and guiding therapy. The diagnosis is confirmed when clonal BMPCs ≥10% or a biopsy-proven bony or extramedullary plasmacytoma are accompanied by at least one MDE, represented by the SLiM-CRAB criteria [31,32].

This systematic method ensures that MM is clearly distinguished from precursor conditions, such as MGUS and smoldering myeloma, which lack end-organ damage or myeloma-specific biomarkers. Precise staging, usually performed with the Revised International Staging System (R-ISS), includes laboratory and cytogenetic data to assess prognosis and guide treatment decisions [8]. Early evaluation by a multidisciplinary team is crucial to improve patient outcomes and prevent irreversible organ damage in those with suspected or confirmed MM [32,33]. The diagnostic criteria and workup, including laboratory testing, imaging, bone marrow confirmation, CRAB features, and SLiM biomarkers, are summarized in Figure 5.

**Figure 5:**
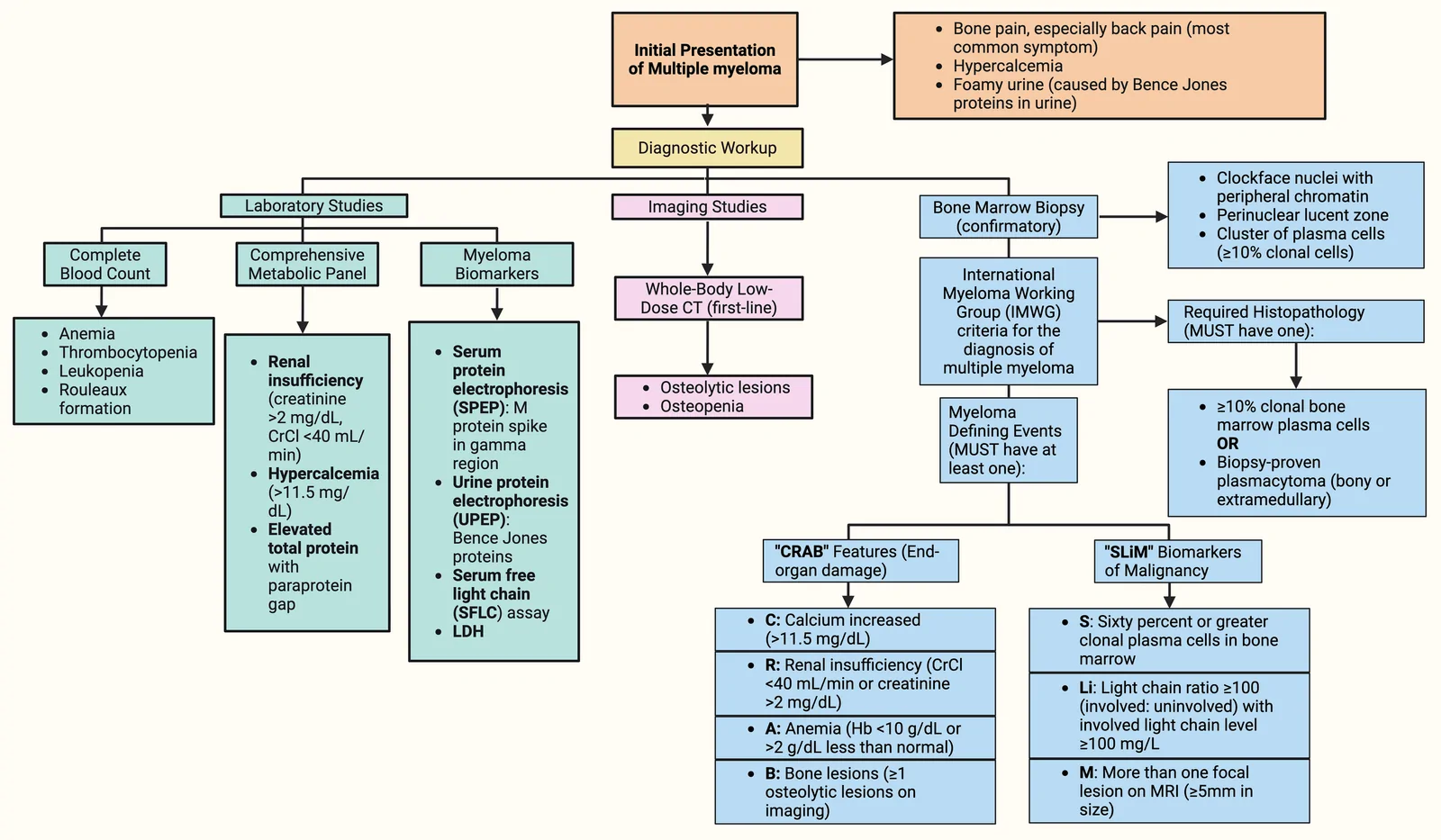
Diagnostic criteria of multiple myeloma. This illustration supports the diagnostic criteria and workflow by visually depicting the major steps used to identify and diagnose multiple myeloma. SPEP: Serum protein electrophoresis; UPEP: Urine protein electrophoresis; sFLC: Serum free light chain; LDH: Lactate dehydrogenase; IMWG: International Myeloma Working Group; MDE: Myeloma-defining event; CRAB: Hypercalcemia, renal insufficiency, anemia, and bone lesions; CrCl: Creatinine clearance; Hb: Hemoglobin; SLiM: ≥60% clonal plasma cells, involved/uninvolved serum free light chain ratio ≥100 provided that the involved free light chain level is ≥100 mg/L, and >1 focal lesion on MRI, with each lesion measuring ≥5 mm. Image credits: Azhar Hussain, Jack L. Moniot, Mohsena Sumaya, David M. Hughes. Created with BioRender.com. This figure is an original author-created illustration. Artificial intelligence was not used in the creation of this figure.

Pharmacologic management of MM

For pharmacists involved in managing MM, several key pharmacotherapy principles are essential to understand. Current frontline treatment is based on anti-CD38 monoclonal antibody regimens, such as daratumumab or isatuximab, in combination with quadruplet therapy. These therapies are preferred for transplant-eligible and select-fit, non-frail transplant-ineligible patients. Treatment selection for patients with MM requires consideration of transplant eligibility, frailty, cytogenetic risk, renal function, and baseline toxicities. Lenalidomide is the preferred maintenance therapy after autologous hematopoietic cell transplantation (HCT), although certain patients with high-risk disease may require a dual-agent maintenance therapy regimen. Renal impairment is common in MM and requires prompt management and appropriate dose adjustments for select medications, such as lenalidomide [34]. Table 2 provides a general overview of primary therapy regimens and clinical considerations, while specific renal dose adjustments and pharmacist-led care strategies are addressed in the subsequent section.

**Table 2:** Overview of frontline pharmacologic management in multiple myeloma. NCCN: National Comprehensive Cancer Network; HCT: Hematopoietic cell transplantation; MM: Multiple myeloma; Dara-VRd: Daratumumab, bortezomib, lenalidomide, and dexamethasone; Isa-VRd: Isatuximab, bortezomib, lenalidomide, and dexamethasone; Dara-Rd: Daratumumab, lenalidomide, and dexamethasone; VRd: Bortezomib, lenalidomide, and dexamethasone; Rd: Lenalidomide and dexamethasone; CrCl: Creatinine clearance; eGFR: Estimated glomerular filtration rate.

Line of Therapy/Setting	NCCN Preferred Regimen(s)	Clinical Considerations	Key Notes
Primary Therapy: HCT Candidate	• Daratumumab/lenalidomide/bortezomib/dexamethasone (Dara-VRd; category 1) • Isatuximab-irfc/bortezomib/lenalidomide/dexamethasone (Isa-VRd; category 1)	• Assess HCT eligibility early • Renal dysfunction and advanced age are not absolute contraindications to transplant • Consider renal function, neuropathy, cytogenetic risk, and frailty	• Autologous HCT is category 1 after primary therapy • Preferred maintenance: lenalidomide • Two-drug maintenance recommended for high-risk MM
Primary Therapy: HCT Deferred or HCT Is Not Indicated	• Daratumumab/lenalidomide/dexamethasone (Dara-Rd; category 1) • Daratumumab/bortezomib/lenalidomide/dexamethasone (Dara-VRd; category 1) for patients <80 years old who are not frail • Isatuximab-irfc/bortezomib/lenalidomide/dexamethasone (Isa-VRd; category 1) for patients <80 years old who are not frail	• Frailty assessment guides treatment intensity • Continue primary therapy until progression, with dose and duration de-escalation as needed • Strongly consider collecting/storing stem cells for future HCT	• Quadruplets are restricted to patients <80 and not frail • Frail patients may need less intensive regimens • Lenalidomide requires renal dosing
Renal Disease Considerations Across Therapy	• Prompt initiation of effective anti-myeloma therapy for myeloma-related kidney injury • Regimens containing bortezomib and high-dose dexamethasone for significant renal impairment • Switching regimens once renal function improves or stabilizes	• Check CrCl/eGFR and adjust medications for renal function • Lenalidomide dosing must be adjusted based on CrCl • Consider renal ultrasound/biopsy when clinically indicated	• Treat hypercalcemia/hyperuricemia • Discontinue nephrotoxins • Dialysis for refractory electrolyte disturbances, uremia, or fluid overload

Key pharmacist interventions: management and prevention

Pharmacists are unique members of the healthcare system, positioned to aid in the prevention, monitoring, and management of renal injury throughout the course of MM. The best prevention of kidney injury in MM is timely intervention with guideline-based therapeutic medications [35]. However, other supportive strategies are critical in preventing renal impairment. One crucial aspect of supportive care includes hydration. Adequate hydration is essential for kidney health in patients with MM, as dehydration may increase light chain precipitation within the renal tubules. Hydration should be individualized based on patient factors such as volume status, renal function, urine output, and comorbidities, with a common urine output goal of 100-150 mL/hr when clinically appropriate [33]. Specifically, adequate hydration should be emphasized for patients experiencing fluid depletion secondary to hypercalcemia. Careful monitoring of patient fluid status is recommended, and patients with anuria may require a clinically appropriate fluid challenge [35,36]. Pharmacists play a pivotal role in educating patients, as they are often a point of contact within the healthcare system. Educating patients about hydration goals and initiating supportive care protocols are crucial to providing proper care, responsibilities that often involve pharmacists.

Another key responsibility of pharmacists is the identification and management of nephrotoxic medications. Medications including NSAIDs, aminoglycosides, contrast agents, angiotensin-converting enzyme inhibitors, and loop diuretics should be avoided when possible in patients with MM-related renal dysfunction [36]. These medications may worsen renal injury in MM, particularly in the setting of myeloma cast nephropathy, which is driven by high levels of immunoglobulin free light chains and their direct nephrotoxic effects [37]. Other potentially harmful exposures to address include alcohol, recreational drugs, tobacco use, and heavy metal exposure. Alcohol and recreational drug use have been associated with CKD progression, while smoking can increase CKD risk through mechanisms including oxidative stress, endothelial dysfunction, glomerulosclerosis, and tubular atrophy [38]. Provider alerts and medication reconciliation allow pharmacists to better manage medication safety, reduce nephrotoxic exposure, and adjust evidence-based therapies accordingly. Pharmacists should also prioritize drug and alcohol screening and utilize smoking cessation tools when interacting with patients. These efforts are especially important in patients with MM, as they are often undergoing intensive therapy while managing renal limitations.

According to studies evaluating MM-associated kidney injury, approximately 10% of patients with MM-related renal impairment may require hemodialysis, and myeloma cast nephropathy is a major cause of dialysis-dependent AKI in this population [37,39]. Therefore, patients with severe AKI coupled with cast nephropathy require urgent reduction of serum free light chains (sFLCs). Because kappa and lambda FLCs weigh approximately 22.5 and 45 kDa, respectively, conventional dialysis and high-flux hemodialysis (HF-HD) have limited ability to clear these molecules due to membrane pore-size restrictions [39]. Recently, there has been ongoing discussion about the effectiveness of extracorporeal techniques in clearing sFLCs [40]. One extracorporeal clearance method historically used to manage cast nephropathy is therapeutic plasma exchange (TPE). Early reports supported the use of TPE to clear sFLCs in MM, given its effectiveness in other paraproteinemias. However, subsequent trials failed to consistently demonstrate clinical benefit, and the role of TPE in myeloma cast nephropathy remains controversial [41].

High-cutoff hemodialysis (HCO-HD) combined with chemotherapy has been investigated as a possible strategy to improve sFLC clearance in patients with MM and AKI. HCO membranes have a cutoff of approximately 45-60 kDa, allowing enhanced removal of both kappa and lambda FLCs [39]. Studies evaluating HCO-HD have shown substantial sFLC clearance, and Xing et al. reported FLC removal rates ranging from 27-72%, with 42-86% of patients recovering renal function and becoming independent of hemodialysis [40]. These findings contributed to the development of randomized controlled trials such as MYRE and EuLITE, which compared HCO-HD with HF-HD. Unfortunately, neither trial showed a statistically significant improvement in dialysis independence within the first three months [40,42]. Notably, MYRE demonstrated higher hemodialysis independence at 6 and 12 months, suggesting that renal recovery from severe AKI in MM may require longer follow-up [40]. However, EuLITE did not support routine use of HCO-HD, and limitations across studies included differences in dialysis filters, chemotherapy regimens, and dialysis protocols [42]. Although HCO-HD can achieve rapid sFLC removal, its effect on clinically meaningful renal outcomes remains uncertain, and it should not replace effective anti-myeloma therapy [39,40]. Additional studies are needed to clarify the optimal role of HCO-HD in patients with MM and renal impairment.

Pharmacists have a significant contribution in the management of MM-related renal dysfunction by coordinating early nephrology referral, monitoring sFLC kinetics to help guide dialysis duration, managing hematologic and electrolyte abnormalities associated with HCO membranes, and ensuring chemotherapy agents such as bortezomib, lenalidomide, and corticosteroids are administered and dosed appropriately to support renal recovery and reduce FLC levels [36,43]. In hospital settings, pharmacists are viewed as medication experts within the healthcare system and have the tools necessary to collaborate with nephrology and hematology specialists to determine the most appropriate extracorporeal technique and therapy for each individual patient.

Beyond acute interventions, patients with MM require long-term renal protection, which includes routine renal monitoring, dose adjustments, and avoidance of nephrotoxins [43]. For example, bisphosphonates can be used to treat myeloma-associated hypercalcemia; however, they are generally not recommended in patients with CrCl <30 mL/min [43,44]. Denosumab may also be used to correct hypercalcemia and can be used in patients with renal impairment but requires close monitoring for hypophosphatemia and hypocalcemia [43]. Other medications may require dose adjustments depending on creatinine clearance. For example, lenalidomide is safe and effective with dexamethasone in renally impaired patients but requires dose adjustment based on renal function [36,44]. Pomalidomide is also safe and effective for relapsed or refractory MM with renal impairment, including patients on dialysis, although the strength of recommendation may vary depending on the degree of renal dysfunction [43]. Dose changes and medication recommendations such as these fall under pharmacists’ responsibilities, as they must ensure appropriate dosing and utilize clinical support tools to flag high-risk prescriptions. Pharmacists must also counsel patients to avoid NSAIDs, maintain adequate hydration, and provide individualized guidance regarding calcium and phosphate intake based on serum levels and treatment requirements [43,44].

Pharmacists have the ability to play a critical role in the management and prevention of kidney injury in patients with MM. Pharmacists must ensure that all patients with MM are counseled on proper hydration and electrolyte management, coordinate with nephrology specialists to determine appropriate extracorporeal techniques and therapies, and manage long-term renal protection to support remission and reduce further renal injury. Examples of commonly used MM therapies requiring renal dose adjustment or careful renal monitoring are summarized in Table 3.

**Table 3:** Renal dosing and monitoring considerations for selected multiple myeloma therapies. CrCl: Creatinine clearance; ESRD: End-stage renal disease; CD38: Cluster of differentiation 38; SLAMF7: Signaling lymphocytic activation molecule family member 7.

Medication	Drug Class	Renal Consideration	When/Why
Ixazomib	Proteasome inhibitor	Dose adjust	Reduce starting dose to 3 mg for CrCl <30 mL/min or ESRD requiring dialysis
Carfilzomib	Proteasome inhibitor	Use caution/monitor	No routine starting renal dose adjustment, but monitor closely for renal toxicity and overall tolerability
Lenalidomide	Immunomodulatory agent	Dose adjust	Adjust dose based on CrCl; renal dosing is required because exposure increases with renal impairment
Pomalidomide	Immunomodulatory agent	Dose adjust in dialysis	Reduce to 3 mg daily in severe renal impairment requiring dialysis; administer after hemodialysis
Daratumumab	Anti-CD38 monoclonal antibody	No renal dose adjustment; monitor combination regimen	Often combined with renally adjusted agents such as lenalidomide
Elotuzumab	SLAMF7 monoclonal antibody	No renal dose adjustment; monitor combination regimen	Often combined with lenalidomide or pomalidomide, which may require renal dose adjustment

Anti-CD38 monoclonal antibodies, such as daratumumab, and SLAMF7 monoclonal antibodies, such as elotuzumab, are generally safe and effective for patients with MM and renal impairment, including those on dialysis. However, these medications are often used in combination regimens that may include agents requiring renal dose adjustment. For example, daratumumab-bortezomib-melphalan-prednisone is safe and effective with CrCl ≥40 mL/min, while daratumumab-lenalidomide-dexamethasone requires CrCl ≥30 mL/min for safe and effective use [43,44]. This general principle may also apply to other MM treatment combinations.

Limitations

This narrative review has several limitations. Selection bias may occur because a risk-of-bias assessment and a formal systematic search or study selection process were not performed. This review also covers select renal complications of MM and does not include all potential manifestations of MM-related kidney disease.

## Conclusions

MM-related kidney disease remains a major complication that significantly impacts patient prognosis, treatment selection, and medication safety. Although renal impairment can progress rapidly, renal sequelae are potentially reversible with early recognition and intervention. Pharmacists are essential members of the healthcare system, playing critical roles in identifying renal risk factors, preventing nephrotoxic exposures, adjusting medications based on renal status, optimizing supportive care, and educating patients on adherence, hydration, and other protective strategies. As treatments and new data in MM evolve, pharmacists must stay current with best practices for renally adjusted therapies and evidence-based care recommendations. Ultimately, collaborative multidisciplinary care involving pharmacists, hematology/oncology specialists, nephrologists, nurses, and patients is necessary to preserve kidney function, reduce treatment-related complications, and improve both renal and overall survival.
